# Knowledge and utilization of the partograph: A cross-sectional survey among obstetric care providers in urban referral public health institutions in northwest and southwest Cameroon

**DOI:** 10.1371/journal.pone.0172860

**Published:** 2017-02-24

**Authors:** Carlson–Babila Sama, Noah F. Takah, Valery K. Danwe, Uzeru Forchu Melo, Therence Nwana Dingana, Fru F. Angwafo

**Affiliations:** 1 Islamic Medicalized Health Centre, Babessi, Cameroon; 2 Galactic Corps Research Group (GCRG), Buea, Cameroon; 3 School of Medicine, University of Glasgow, Glasgow, United Kingdom; 4 Saint Blaise Catholic Hospital, Bamenda, Cameroon; 5 Department of Obstetrics and Gynaecology, Buea Regional Hospital, Buea, Cameroon; 6 Catholic General Hospital, Njinikom, Cameroon; 7 Gynaeco-Obstetric and Paediatric Hospital, Yaoundé, and Department of Surgery, University Teaching Hospital Yaoundé I, Yaoundé, Cameroon; University of Ottawa, CANADA

## Abstract

**Background:**

The enormous challenge to maternal well-being with associated maternal wastages during labour has remained an unsurmountable problem in Cameroon which reflects the current high maternal mortality rate. Evidence abounds that cost-effective and affordable health interventions like the use of the partograph will contribute to curb the alarming number of intrapartum maternal deaths. However, little is known about the level of knowledge and utilization of this simple life-saving tool in the North–and South–West Regions, Cameroon.

**Methods:**

Using a self-administered structured questionnaire, a cross-sectional study was conducted from January 4^th^–March 25^th^ 2016 among non-physician obstetric care providers (OCPs) across urban public health institutions in these regions. Logistic regression models were used to identify factors associated with good knowledge and routine utilization of the partograph.

**Results:**

Of the 79 eligible participants, 71 (89.9%) took part in the study. The mean age of the respondents was 37.9±10.0 years with majority being female (85.9%). Less than one-third (29.6%) of the respondents had good knowledge on the partograph and only 23 (32.4%) routinely used it in monitoring labour. OCPs working in Maternal and Infant Welfare Clinics were about 4 times more likely than those working in Regional/District Hospitals to have good knowledge on the partograph [AOR = 3.88 (95% CI:1.07–14.04)], *p* = 0.04. Little or no knowledge of the partograph and poor staff strength in the study centres were factors militating against its routine use.

**Conclusions:**

The knowledge and use of the partograph in this study is sub-optimal. Regular in-service training of OCPs superimposed with periodic workshops and seminars, provision of reasonable staff numbers, and mandatory institutional policies on routine use of the partograph are recommended as vital first steps towards ensuring the safety of women in labour in the North–and South–West Regions of Cameroon.

## Background

In 2015, global estimates from the World Health Organisation (WHO) showed that there were 216 maternal deaths per 100,000 live births. Approximately 99% of these global maternal deaths occurred in developing regions with sub-Saharan Africa accounting for roughly 66% [1]. In Cameroon, the current maternal mortality ratio (MMR) stands at 596 deaths per 100,000 live births, which is among the highest in the world, with a resultant failure to attain the Millennium Development Goal 5 (MDG5) [1]. In a bid to build on the momentum generated by MDG5, the WHO has laid out a transformative new agenda for maternal health as part of the Sustainable Development Goals (SDGs) whose primary objectives are to reduce the global MMR to less than 70 per 100,000 live births by 2030 (SDG3.1) [2], and that no country should have a MMR greater than 140 per 100,000 live births by 2030 [3]. To achieve these objectives, proven interventions to prevent such deaths, including improving the knowledge of obstetric care providers (OCPs) in midwifery skills should be promoted in order to ensure skilled attendance during labour and delivery of the parturient woman.

Maternal mortality resulting from prolonged and obstructed labour is preventable, and there are convincing reports that acquisition of adequate knowledge and proper utilization of the partograph would culminate in a remarkable reduction in its incidence, which currently constitutes about 8–10% of maternal deaths [4–8]. The partograph (S1 Fig) is a simple graphic recording of labour and salient conditions of the mother and foetus against time in hours [8]. It serves as a one page–visual summary of the relevant details of labour including but not limited to: foetal heart rate; cervical dilatation; foetal descent; uterine contraction; maternal blood pressure and drugs used during labour [9,10]. The principle of the partograph is based on the fact that, the rate of cervical dilation should not be slower than 1cm/hour during the active phase of labour [9]. Proper utilization of the partograph in accordance with standard protocol [10], with keen attention to the alert and action lines allows for timely identification and diagnosis of pathologic labour and thus aid in guiding timely decision-making regarding the necessary interventions.

Following recommendations from the WHO, the Maternal and Neonatal Health program promotes the routine use of the partograph to monitor the progress of labour in all settings [3,5,6,11]. Therefore, OCPs at all levels are expected to know how to correctly use and interpret the partograph, which will in turn significantly reduce the sequelae of maternal and foetal wastages. Although the partograph is a simple and inexpensive tool which has been proven to effectively prevent maternal deaths and the ensuing complications due to obstructed or prolonged labour, it is not as widely implemented as it should be [12,13]. Furthermore, studies from Ethiopia [5,13,14], Nigeria [4,6–8,15], Malawi [16], Tanzania [10] and Kenya [17] have reported significant gaps in the knowledge and application of this tool.

In Cameroon, primary obstetric care services are mostly rendered by midwives and nurses of diverse training. Limited studies have been done to assess their knowledge and utilization of the partograph [18,19]. An understanding of the current level of knowledge and factors associated with the use of the partograph among these OCPs will be essential in informing policy on obstetric care improvement through the provision of guided educational training programs and updates on adequate management and surveillance of labour in order to further empower them in safe motherhood practices.

## Methods

### Study design, setting and population

This was an analytical cross-sectional study from January 4^th^ 2016 to March 25^th^ 2016 among non-physician obstetric care providers in referral public health institutions in the chief cities of Anglo-Saxon Cameroon (South-West and North-West Regions). As of 2010, these two regions have a combined population of over 3,188,981 inhabitants. This figure is a projection derived from the November 2005 population census. These regions comprise many public, private/lay private and faith–based health institutions. In each urban town in the South-West Region (Limbe, Buea, Kumba) and North-West Region (Bamenda), the 2 main reference public health institutions were included; the Regional/District Hospitals (R/DH) and the Maternal and Infant Welfare Clinics (MIWC). These referral health institutions offer obstetric services including family planning methods, pre-, ante- and post-natal consultations as well as monitoring labour and conducting deliveries either vaginally or via a caesarean section. The target population was all consenting midwives and nurses working in the maternity units of these institutions.

### Study procedure and data collection

A structured self-administered questionnaire was used for data collection. It was designed to document respondents’ socio-demographic and professional characteristics, knowledge of partograph, and their attitude towards its utilization. Reliability and validity of the questionnaire were ensured through consultation with experts and by adequate review of several relevant literatures [7,8,14]. It was pre-tested in a pilot study on 16 OCPs (5 midwives and 11 nurses) working in the maternity units in other public health institutions which were not part of the actual study. Findings from the pre-test were used to modify the questionnaire in terms of clarifying the questions. Five clinical year medical students facilitated the data collection process. They had received prior training about the aim of the study, procedures, and data collection techniques. Progress was reviewed and research assistants re-trained periodically in each coordinating centre during this phase. The principal investigators assisted the supervision process.

### Scoring of knowledge of OCPs

A knowledge scoring system was developed in order to produce a more objective assessment of the OCPs knowledge of the partograph. Based on this system, a “knowledge score” was obtained via assessment with 24 composite questions (S1 File) [8]. Based on the overall knowledge scores, respondents with scores within the range (1–8) were categorized as having “poor knowledge”; those who scored between 9 and 16 were considered as having “fair knowledge” while those with scores ranging 17 to 24 were categorized as having “good knowledge”. In order to investigate the factors associated with good knowledge, the population was split into two groups; OCPs with “poor-to-fair knowledge (score < 17)” and those with “good knowledge (score ≥ 17)”. Similarly, we further divided the population into two groups; “routinely” = (always) and “not routinely” = (never, rarely, sometimes, often) in order to investigate the factors associated with utilization of the partograph.

### Data analysis

The returned questionnaires were visually checked for completeness, coded and entered into a Microsoft excel spreadsheet. Once the entry was completed, the data was exported to IBM-SPSS statistical software v.20 for Windows (SPSS Inc., Chicago, IL) for analysis. Frequency run and double data entry on 10% of the questionnaires was performed to check data entry errors. Categorical variables were summarized as counts and percentages while continuous variables were summarized as means and standard deviations. Chi square test (χ^2^) was used to test the significance of association between categorical variables. The relationship between selected independent variables and the respondents’ level of knowledge and utilization of the partograph were explored using binary logistic regression analysis at 95% Confidence Interval (CI). While adjusting for age and gender, all variables with a *p*–value ≤0.25 in bivariate analyses were included in the multivariate model [20]. The level of significance was set at *p*-value <0.05.

### Ethics statement

Ethical approval was obtained from the administrative and ethical committee of the respective health institutions and also from the institutional review boards of the regional delegation of the ministry of health for the South- and North-West Regions. Written informed consent was obtained from each participant before enrolment into the study. Confidentiality of participants was maintained via coding of questionnaires and this study adhered to the declaration of Helsinki.

## Results

### Socio-demographic characteristics of study participants

During the study period, a total of 82 OCPs were working in the maternity units of these institutions, amongst which 79 consented to the study and were administered the questionnaires. Seventy-six questionnaires were returned, giving a response rate of 96.2%. Of these, 5 were disqualified due to incompleteness. In total, 71 properly filled questionnaires were analysed. The mean age of the participants was 37.9 ± 10.0 years and ranged from 22–58 years. There was a female predominance (85.9%) and most (63.4%) of the participants were nurses. Forty-four (62%) were married and over half (52.1%) of the participants worked in MIWCs. The mean length of practice was 10.34 ± 8.5 years (range: 1–35 years), with over a third (35.2%) practicing for less than 6 years. Table 1 summarizes the socio-demographic profile of the study participants.

**Table 1:** Sociodemographic characteristics of study participants.

Variables	R/DH n (%)	MIWC n (%)	Total n (%)
**Age (years)**			
20–29	8 (23.5)	12 (32.5)	20 (28.2)
30–39	12 (35.3)	8 (21.6)	20 (28.2)
40–49	9 (26.5)	6 (16.2)	15 (21.1)
≥50	5 (14.7)	11 (29.7)	16 (22.5)
Total	34 (47.9)	37 (52.1)	71 (100)
**Gender**			
Male	4 (11.8)	6 (16.1)	10 (14.1)
Female	30 (88.2)	31 (83.9)	61 (85.9)
Total	34 (100)	37 (100)	71 (100)
**Marital status**			
Married	26 (76.5)	18 (48.6)	44 (62)
Single*	8 (23.5)	19 (51.4)	27 (38)
Total	34 (100)	37 (100)	71 (100)
**Religion**			
Christian	29 (85.3)	36 (97.3)	65 (91.5)
Muslim	2 (5.9)	0 (0)	2 (2.8)
Others	3 (8.8)	1 (2.7)	4 (5.6)
Total	34 (100)	37 (100)	71 (100)
**Professional qualification**			
Midwife	12 (35.3)	14 (37.8)	26 (36.6)
Nurse	22 (64.7)	23 (62.2)	45 (63.4)
Total	34 (100)	37 (100)	71 (100)
**Professional tenure (years)**			
1–5	12 (35.4)	13 (35.2)	25 (35.2)
6–10	10 (29.4)	11 (29.7)	21 (29.5)
11–15	6 (17.6)	4 (10.8)	10 (14.1)
16–20	3 (8.8)	3 (8.1)	6 (8.5)
> 20	3 (8.8)	6 (16.2)	9 (12.7)
Total	34 (100)	37 (100)	71 (100)

*Single = Unmarried, Divorced, Widow/Widower

### Awareness and knowledge of the partograph

Table 2 details the respondents’ knowledge on the definition of the partograph, its use as a preventive tool and the normal graphic patterns. About one-third (33.8%) of the respondents were unable to identify the correct definition of the partograph as a simple graphic recording of labour and salient conditions of the mother and foetus against time in hours. Similarly, 29.6% regarded it as a complex tool with pictorial overview of labour for use by midwives. The OCPs’ knowledge on the partograph as a preventive tool showed that 60.6%, 78.9% and 64.8% of respondents thought its use will reduce maternal morbidity, reduce maternal mortality and increase the efficiency of those attending to women in labour respectively.

**Table 2:** Knowledge on the partograph among obstetric care providers in public hospitals in Southwest and Northwest Regions, Cameroon 2016.

**Respondents’ knowledge on the definition of the partograph**					
**Variables**	**R/DH n (%)**	**MIWC n (%)**	**Total n (%)**	**χ**^**2**^	***p*-value**
**A chart developed by midwives in developed countries to monitor labour**					
Yes	10 (29.4)	5 (13.5)	15 (21.1)	3.23	0.199
No	15 (44.1)	23 (62.2)	38 (53.5)		
Don’t know	9 (26.5)	9 (24.3)	18 (25.4)		
**A complex tool with pictorial overview of labour for use by midwives**					
Yes	12 (35.3)	9 (24.3)	21 (29.6)	3.19	0.203
No	14 (41.2)	23 (62.2)	37 (52.1)		
Don’t know	8 (23.5)	5 (13.5)	13 (18.3)		
**A chart for monitoring labour by doctors**					
Yes	7 (20.6)	1 (2.7)	8 (11.3)	9.57	0.008
No	12 (35.3)	25 (67.6)	37 (52.1)		
Don’t know	15 (44.1)	11 (29.7)	26 (36.6)		
**A simple graphic recording of labour and salient conditions of the mother and fetus against time in hours**					
Yes	21 (61.8)	26 (70.3)	47 (66.2)	4.61	0.100
No	4 (11.8)	0	4 (5.6)		
Don’t know	9 (26.4)	11 (29.7)	20 (28.2)		
**Respondents’ knowledge of partograph as a preventive tool**					
**Will reduce maternal morbidity**					
Yes	18 (52.9)	25 (67.6)	43 (60.6)	1.74	0.418
No	3 (8.8)	3 (8.1)	6 (8.4)		
Don’t know	13 (38.2)	9 (24.3)	22 (31.0)		
**Will reduce maternal mortality**					
Yes	27 (79.4)	29 (78.4)	56 (78.9)	0.72	0.696
No	5 (14.7)	4 (10.8)	9 (12.7)		
Don’t know	2 (5.9)	4 (10.8)	6 (8.5)		
**Will reduce neonatal morbidity**					
Yes	19 (55.9)	24 (64.9)	43 (60.6)	0.64	0.724
No	4 (11.8)	3 (8.1)	7 (9.9)		
Don’t know	11 (32.4)	10 (27.1)	21 (29.5)		
**Will reduce neonatal mortality**					
Yes	24 (70.6)	27 (73.0)	51 (71.8)	0.05	0.975
No	3 (8.8)	3 (8.1)	6 (8.5)		
Don’t know	7 (20.6)	7 (18.9)	14 (19.7)		
**Will increase the efficiency of those attending to women in labour**					
Yes	23 (67.6)	23 (62.2)	46 (64.8)	0.34	0.843
No	7 (20.6)	8 (21.6)	15 (21.1)		
Don’t know	4 (11.8)	6 (16.2)	10 (14.1		
**Is mandatory for improved quality of care of a woman in labour**					
Yes	22 (64.8)	24 (64.9)	46 (64.8)	0.03	0.982
No	6 (17.6)	6 (16.2)	12 (16.9)		
Don’t know	6 (17.6)	7 (18.9)	13 (18.3)		
**Respondents’ knowledge of detailed graphic representation of normal labour**					
**Variables**	**R/DH n (%)**	**MIWC n (%)**	**Total n (%)**	**χ**^**2**^	***p*-value**
**I. Functions of the alert line**					
**The graph on the partograph should fall to the left of the alert line**					
Yes	18 (52.9)	12 (32.4)	30 (42.3)	6.13	0.047
No	5 (14.7)	15 (40.5)	20 (28.2)		
Don’t know	11 (32.4)	10 (27.1)	21 (29.5)		
**The graph on the partograph should fall on the alert line**					
Yes	14 (41.2)	22 (59.5)	36 (50.7)	3.71	0.156
No	12 (35.3)	6 (16.2)	18 (25.4)		
Don’t know	8 (23.5)	9 (24.3)	17 (23.9)		
**The graph on the partograph should fall to the right of the alert line**					
Yes	2 (5.9)	7 (18.9)	9 (12.7)	4.06	0.048
No	19 (55.9)	22 (59.5)	41 (57.7)		
Don’t know	13 (38.2)	8 (21.6)	21 (29.6)		
**II. Functions of the action line**					
**Indicates appropriate action must be taken**					
Yes	22 (64.7)	28 (75.7)	50 (70.4)	4.66	0.097
No	4 (11.8)	0 (0)	4 (5.6)		
Don’t know	8 (23.5)	9 (24.3)	17 (24.0)		
**Time for the woman to be adequately re-assessed for appropriate intervention**					
Yes	8 (23.5)	8 (21.6)	16 (22.5)	0.11	0.943
No	17 (50.0)	20 (54.1)	37 (52.1)		
Don’t know	9 (26.5)	9 (24.3)	18 (25.4)		
**Continuous observation till delivery**					
Yes	10 (29.4)	10 (27.0)	20 (28.2)	0.84	0.657
No	15 (44.1)	20 (54.1)	35 (49.3)		
Don’t Know	9 (26.5)	7 (18.9)	16 (22.5)		
**Respondents’ knowledge on characteristics of normal labour**					
**Variables**	**R/DH n (%)**	**MIWC n (%)**	**Total n (%)**	**χ**^**2**^	***p*-value**
**Three/four contractions in every 10 minutes is normal**					
Yes	27 (79.4)	30 (81.1)	57 (80.3)	0.46	0.796
No	1 (2.9)	2 (5.4)	3 (4.2)		
Don’t know	6 (17.7)	5 (13.5)	11 (15.5)		
**Minimum duration of a strong contraction is 40 seconds**					
Yes	22 (64.7)	24 (64.9)	46 (64.8)	0.16	0.923
No	2 (5.9)	3 (8.1)	5 (7.0)		
Don’t know	10 (29.4)	10 (27.0)	20 (28.2)		
**You require 10 minutes to effectively assess adequacy of contractions**					
Yes	19 (55.9)	23 (62.2)	42 (59.2)	1.19	0.553
No	3 (8.8)	5 (13.5)	8 (11.3)		
Don’t know	12 (35.3)	9 (24.3)	21 (29.5)		
**Progress of labour is assessed by the degree of cervical dilatation and descent of the presenting part**					
Yes	28 (82.4)	35 (94.6)	63 (88.7)	5.23	0.073
No	0 (0)	1 (2.7)	1 (1.4)		
Don’t know	6 (17.6)	1 (2.7)	6 (17.6)		
**Labour is prolonged when it lasts more than 12 hours**					
Yes	17 (50)	27 (73.0)	44 (62.0)	4.68	0.096
No	8 (23.5)	3 (8.1)	11 (15.5)		
Don’t know	9 (26.5)	7 (18.9)	16 (22.5)		

Knowledge of graphic representation of normal labour was poor. Only 42.3% correctly stated that in the normal progress of labour, the graph/plot on the partograph should fall to the left of the alert line. Less than a quarter (22.5%) of the participants knew that the action line indicates that it is time for the woman to be adequately re-assessed for appropriate intervention. As many as 20 (28.2%) and 16 (22.5%) incorrectly responded “yes” and “don’t know” respectively to the question item that the action line indicates that the woman should be continuously observed till delivery.

With respect to the characteristics of contractions and progress of labour, over 10% did not agree that the progress of labour is assessed by the degree of cervical dilatation and descent of the presenting part. Slightly more than half (59.2%) agreed that 10 minutes is required to effectively assess the adequacy of contractions.

OCPs knowledge about assessment made with the partograph during labour showed that most respondents indicated that the partograph can be used in detecting prolonged labour (98.6%), poor progress of labour (97.2%), need for caesarean section (93%) while only 22.5% knew about maternal dehydration as an assessment that could be inferred from the partograph (Table 3).

**Table 3:** Knowledge on assessment inferred from the partograph among obstetric care providers in public hospitals in Southwest and Northwest Regions, Cameroon 2016.

Variables	R/DH n (%)	MIWC n (%)	Total n (%)	χ^2^	*p*-value
**Prolonged labour**					
Yes	33 (97.1)	37 (100)	70 (98.6)	1.10	0.479
No	1 (2.9)	0 (0)	1 (1.4)		
Don’t know	0 (0)	0 (0)	0 (0)		
**Obstructed labour**					
Yes	26 (76.5)	29 (78.4)	55 (77.5)	1.37	0.503
No	3 (8.8)	1 (2.7)	4 (5.6)		
Don’t know	5 (14.7)	7 (18.9)	12 (16.9)		
**Poor progress of labour**					
Yes	33 (97.1)	36 (97.3)	69 (97.2)	0.004	0.952
No	0 (0)	0 (0)	0 (0)		
Don’t know	1 (2.9)	1 (2.7)	2 (2.8)		
**Inefficient uterine contraction**					
Yes	26 (76.5)	30 (81.1)	56 (78.9)	3.49	0.174
No	3 (8.8)	0 (0)	3 (4.2)		
Don’t know	5 (14.7)	7 (18.9)	12 (16.9)		
**Suspected foetal distress**					
Yes	28 (82.4)	29 (78.4)	57 (80.3)	0.32	0.854
No	1 (2.9)	2 (5.4)	3 (4.2)		
Don’t know	5 (14.7)	6 (16.2)	11 (15.5)		
**Abnormal FHR**					
Yes	30 (88.2)	29 (78.4)	59 (83.1)	5.50	0.064
No	2 (5.9)	0 (0)	2 (2.8)		
Don’t know	2 (5.9)	8 (21.6)	10 (14.1)		
**Satisfactory progress of labour**					
Yes	28 (82.4)	32 (86.5)	60 (84.5)	1.14	0.565
No	1 (2.9)	0 (0)	1 (1.4)		
Don’t know	5 (14.7)	5 (13.5)	10 (14.1)		
**Need for labour augmentation**					
Yes	26 (76.5)	29 (78.4)	55 (77.5)	0.04	0.982
No	1 (2.9)	1 (2.7)	2 (2.8)		
Don’t know	7 (20.6)	7 (18.9)	14 (19.7)		
**Need for Caesarean delivery**					
Yes	32 (94.2)	34 (91.9)	66 (93.0)	0.27	0.875
No	1 (2.9)	1 (2.7)	2 (2.8)		
Don’t know	1 (2.9)	2 (5.4)	3 (4.2)		
**Dehydration in mother**					
Yes	9 (26.5)	7 (18.9)	16 (22.5)	0.58	0.748
No	14 (41.2)	17 (46.0)	31 (43.7)		
Don’t know	11 (32.3)	13 (35.1)	24 (33.8)		

Overall, the knowledge scores ranged between 2 and 20, with a mean score of 12.8 (SD = 5.7). Up to 22 (31.0%) of respondents had poor knowledge, the majority 28 (39.4%) had moderate knowledge while less than one-third (29.6%) of the respondents were found to have good knowledge of the partograph (Table 4).

**Table 4:** Overall knowledge scores on the partograph among obstetric care providers in public hospitals in Southwest and Northwest Regions, Cameroon 2016.

	R/DH n (%)	MIWC n (%)	Total n (%)	*p*-value (χ^2^)	Mean ± SD	*p*—value (ANOVA)
**Good**	6 (28.6)	15 (71.4)	21 (29.6)	0.11	18.9 ± 0.6	<0.0001
**Fair**	16 (57.1)	12 (42.9)	28 (39.4)		14.3 ± 1.4	
**Poor**	12 (54.5)	10 (45.5)	22 (31.0)		5.3 ± 2.2	
Total	34 (47.9)	37 (52.1)	71 (100)		12.8 ± 5.7	

### Factors militating against routine use of the partograph

All participants 71 (100%) knew what the partograph was. However, a significantly low proportion (7.0%) had never used it in the management of labour. Most (90.1%) of the participants had received prior training on the use of the partograph. Of these, almost all midwives [25/26 (96.2%)] had received prior training, while only 86.7% (39/45) of nurses had been previously trained. Of all respondents, only 23 (32.4%) of them reportedly used the modified WHO partograph routinely (always) to monitor women in labour. Amongst others, the main reasons advanced for the non-routine utilisation of this tool by these OCPs were; “little or no knowledge” (35.4%) on how to complete it and “shortage of staff” (31.3%). Four (*n* = 48, 8.3%) of the participants felt it is easier to manage labour without use of the partograph (Table 5). All respondents (*n* = 71, 100%) expressed the desire to be trained/re-trained on its use.

**Table 5:** Utilization of the partograph among obstetric care providers in public hospitals in Southwest and Northwest Regions, Cameroon 2016.

Variable	R/DH n (%)	MIWC n (%)	Total n (%)
**Frequency of usage, n = 71**			
Always	13 (56.5)	10 (43.5)	23 (32.4)
Often	10 (32.3)	21 (67.7)	31 (43.7)
Sometimes	7 (77.8)	2 (22.2)	9 (12.7)
Rarely	3 (100)	0 (0)	3 (4.2)
Never	1 (20)	4 (80)	5 (7.0)
**Reasons for non-routine usage, n = 48**			
Little or no knowledge	7 (41.2)	10 (58.8)	17 (35.4)
Shortage of staff	5 (33.3)	10 (66.7)	15 (31.3)
Time-consuming	6 (66.7)	3 (33.3)	9 (18.8)
Easier to manage labour without use of the partograph	2 (50)	2 (50)	4 (8.3)
Non-availability of partograph	1 (33.3)	2 (66.7)	3 (6.2)

### Determinants of good knowledge and routine utilization of the partograph

Table 6 shows some selected professional characteristics and other variables which were tested for association with good knowledge and routine utilization of the partograph by these obstetric care providers. On crude logistic regression analysis, nurses where about 3 times more likely to have good knowledge on the partograph than midwives [COR = 3.33 (95% CI: 0.98–11.35)]. This finding was marginally significant (*p* = 0.054). Similarly, being single [COR = 3.11 (95% CI: 1.10–8.91)] and working in the MIWCs [COR = 3.18 (95% CI: 1.06–9.55)] were significantly associated with good knowledge on bivariate logistic regression analysis. However, only working in MIWCs remained significantly (*p* = 0.039) associated with good knowledge on multivariate logistic regression analysis [AOR = 3.88 (95% CI: 1.07–14.04)]. None of the variables tested was found to be associated with routine utilization of the partograph in univariable logistic regression models (S1 File).

**Table 6:** Determinants of good knowledge on the partograph among obstetric care providers in public hospitals in Southwest and Northwest Regions, Cameroon 2016.

	Poor-to-Fair n (%)	Good n (%)	COR (95% CI)	*p*-value	AOR (95% CI)	*p*-value
**Age**						
≤ 36 years	23 (62.2)	14 (37.8)	1		1	
> 36 years	27 (79.4)	7 (20.6)	0.43 (0.14–1.23)	0.116	0.34 (0.09–1.35)	0.130
**Gender**						
Male	8 (80.0)	2 (20.0)	1		1	
Female	42 (68.9)	19 (31.1)	1.81 (0.35–9.34)	0.479	4.18 (0.67–26.04)	0.126
**Marital Status**						
Married	35 (79.5)	9 (20.5)	1		1	
Single	15 (55.6)	12 (44.4)	**3.11 (1.10–8.91)**	0.035*	1.82 (0.51–6.52)	0.359
**Type of Institution**						
R/D hospital	28 (82.4)	6 (17.6)	1		1	
MIWC	22 (59.5)	15 (40.5)	**3.18 (1.06–9.55)**	0.039*	**3.88 (1.07–14.04)**	0.039*
**Professional Qualification**						
Midwife	22 (84.6)	4 (15.4)	1		1	
Nurse	28 (62.2)	17 (37.8)	3.33 (0.98–11.35)	0.054	3.63 (0.96–13.73)	0.057
**Previous training**						
Yes	44 (68.8)	20 (31.2)	2.72 (0.91–5.23)	0.367		
No	6 (85.7)	1 (14.3)	1			
**Years of experience**						
< 10 years	25 (65.8)	13 (34.2)	1			
≥ 10 years	25 (75.8)	8 (24.2)	0.62 (0.22–1.74)	0.361		

* = statistically significant at 95% CI, *p* < 0.05; 1 = reference

## Discussions

In Cameroon, midwives and nurses constitute the bulk of skilled birth attendants; thus, it is imperative that these obstetric care providers (OCPs) be knowledgeable in partographic monitoring of the progress of labour if they are to significantly contribute in reducing the country’s high MMR.

Overall, about one-third (31.0%) of the respondents had poor knowledge on the partograph, which is significantly higher than the 8.7% reported in public health institutions in Addis Ababa [14]. Most (39.4%) had fair knowledge while only a little higher than a quarter (29.6%) of our participants were found to have good knowledge on the partograph. This finding is similar to that of Oladapo et al in Nigeria [21] where less than one-third of the respondents had good knowledge; it’s lower than, but somewhat comparable to the 39.0% observed by Yisma et al [14]. Abebe and colleagues [5] had also reported a lower but comparative lack of knowledge in a study where only 26.6% of health professionals in the Amhara region in Ethiopia had good knowledge on the partograph. Furthermore, our respondents lacked detailed knowledge of the component parts of the partograph vis-à-vis the normal labour tracing and its relationship to the alert and action lines. Knowledge on the characteristics of a good contraction was equally unsatisfactory. These findings fall in line with several other studies [4,5,7,8,14] where it has been shown that most OCPs are unable to correctly identify and explain the functions of the component parts of the partograph including the alert and action lines. Consequently, it brings to light the dire need for very urgent strategies aimed at improving knowledge levels of these OCPs on the partograph via continuous training and retraining programs in order to maximize its proper use.

In this study, more females (31.1%) had good knowledge on the partograph than males (20.0%). However, this observation was not statistically significant. It could be explained, in part, by the fact that females are closer to obstetric information and are more likely to become midwives than males [5]. The higher numbers of females in this study may also be a contributory factor. Interestingly, and in contradiction to several studies [5,7,15,21], nurses were more likely [AOR = 3.63 (95% CI: 0.96–13.73)] to have an overall good knowledge on the partograph than midwives. Given that an overall higher percentage of midwives (96.2%) had prior training on the partograph compared to nurses (86.7%); this marginally significant finding (*p* = 0.057) puts into question the quality of training midwives undergo with respect to the partograph as obstetric care is their major subject in preservice education; thus are expected to have a better understanding of the tool than nurses. Being single had higher odds of having good knowledge of the partograph, though it lost its significance on multivariate logistic regression models. After adjusting for possible confounders, the type of health facility where obstetric care providers were working was significantly associated with good knowledge; OCPs working in MIWCs were about four times more likely to have good knowledge than those working in R/DHs. This can be explained by the fact that, MIWCs are somewhat more specialized units in obstetric care than R/DHs that cater for a wider range of pathologies across the general population. Thus, the maternity staffs in MIWCs may be conscious of the fact that their centres are expected to provide better obstetric services to the pregnant woman than other health institutions.

Almost two decades ago, prior to the implementation of the MDGs, Umezulike and collaborators [22] had identified lack of detailed knowledge of the partograph among medical personnel in Enugu, Nigeria. They also noted that only about 25% of these personnel routinely employed the partograph in monitoring labour. Sadly enough, in the post-MDG era and the prime of the SDGs, the situation has not changed that much. Though all of our participants knew what the partograph was, only one-third (32.4%) of them reportedly used it routinely to monitor the progress of labour. This conforms to more recent reports from similar studies across varied African settings which have confirmed the low utilization of this cost-effective, labour-monitoring chart [5–8,10,14–17,23]. Inadequate knowledge and poor utilization of this tool could be part of the reasons accounting for the high maternal mortality in Cameroon and most third world nations. This necessitates the need for hospital administrators to implement policies which bind every OCP to routinely use the partograph in accordance with WHO recommendations and the safe motherhood initiative [11].

We noted that only 6.3% of respondents who used the partograph stated its unavailability as a reason for its non-routine utilization. This is in contrast to related studies in Nigeria where higher rates; 30.3% [15], 58.6% [8] and 70% [4] of non-availability of the partograph have been reported. However, our study seems to agree with many others [4–8,15] that “little or no knowledge” remains a significant barrier to its effective utilization. This reiterates the invaluable necessity for regular pre-service and on-job training of these OCPs on use of the partograph to ensure safety of the parturient woman. Some authors [4,8,15,21,24] have also identified shortage of staff as a major setback in most health institutions, a finding which corroborated with ours. They opined that the few OCPs on duty are overwhelmed with so much work on a shift such that little or no attention is paid to some important and routine professional details [4,15,24]. This assertion may be applicable to this study as the number of maternity based-OCPs sampled was found to be too small to serve the ever growing population of these regions. It is probably as a result of staff shortages that some OCPs consider the time spent in completing the partograph to be unduly wasted, especially when they do not appreciate the benefits it brings to a woman in labour [24]. Thus, as suggested by some studies [4,15], and by direct implication; the more OCPs assigned per shift, the more likely it is that they will complete the partograph during labour. This therefore informs the need for public health authorities to draft appropriate measures that will ensure the recruitment of more obstetric care providers in public health facilities so as to improve efficiency in partographic labour monitoring.

Almost 50 years since the introduction of the partograph by Philpott [25], it remains a disturbing reality that OCPs across developing nations which have poor maternal mortality indices still lack adequate knowledge on this efficacious life-saving tool with a concomitant low utilization. This should be regarded as a national emergency and calls for a thorough relook into the existing health policies as well as OCP training guidelines. It is hoped that this study will form the basis not only for further research on the partograph and its correlates, but also provoke inquiries into other causes of maternal mortality with committed efforts geared towards enforcement of concrete solutions. In so doing, Cameroon will stand a better chance to stem the tide of maternal morbidity/mortality and neonatal demise during labour.

This study was not conducted within the confines of an epidemiologically representative sample, as such; the small sample size may render estimates as well as associations between dependent and independent variables unstable and therefore undetected, thus a limitation. Also, obstetric care providers in public health centres, private and faith–based health institutions, as well as those in rural public health institutions within these regions were not included. Thus, generalization of the findings should be made with caution. Furthermore, there is the tendency for social desirability bias whereby respondents may be inclined towards giving answers they feel is desirable to the researchers. Despite these limitations, the findings of this study will be useful to health care professionals and policy makers. It may be regarded as a window that provides a glimpse into current knowledge and utilization of the partograph; and are the first steps towards a larger study that will cover all health facilities within the regions and eventually nationwide.

## Conclusion

Whilst maternal mortality remains a serious public health problem in Cameroon, the obstetric care providers’ knowledge and utilization of the partograph in monitoring labour was generally poor. If the state is to achieve its government’s agenda to reduce maternal mortality by 25% in 2018 as well as to be on the roadmap towards attaining the WHO’s SDG3 by 2030, then, amongst others, focused attention must be tilted towards the partograph. All OCPs should be subjected to intensive pre-service training reinforced with periodic on-job refresher courses via unit presentations, seminars, and workshops on use of the partograph. It is imperative that the persisting problem of staff shortages be prioritized and urgently addressed. The hospital administrators should make this simple, cost-effective, labour-monitoring chart readily available to these OCPs at all times and make its routine use mandatory in all patients admitted with a diagnosis of labour.

## Supporting information

S1 Fig Sample of WHO modified partograph. (TIF)

S1 File Criteria for the partograph knowledge score and factors associated with routine utilization of the partograph. (DOCX)

S2 File Populated STROBE checklist. (DOC)

S3 File Data collection sheet. (DOCX)

S4 File Minimal dataset. (SAV)

## Abbreviations

AOR: Adjusted Odds Ratio
CI: Confidence Interval
COR: Crude Odds Ratio
MIWC: Maternal and Infant Welfare Clinic
MMR: Maternal Mortality Rate
OCP: Obstetric Care Provider
OR: Odds Ratio
R/DH: Regional/District Hospital
SD: Standard Deviation
WHO: World Health Organization
