# Supplementary figures and images for: Knowledge and utilization of the partograph: A cross-sectional survey among obstetric care providers in urban referral public health institutions in northwest and southwest Cameroon
Source: PLoS One. 2017 Feb 24;12(2):e0172860. doi: 10.1371/journal.pone.0172860 (PMC5325583; doi:10.1371/journal.pone.0172860)

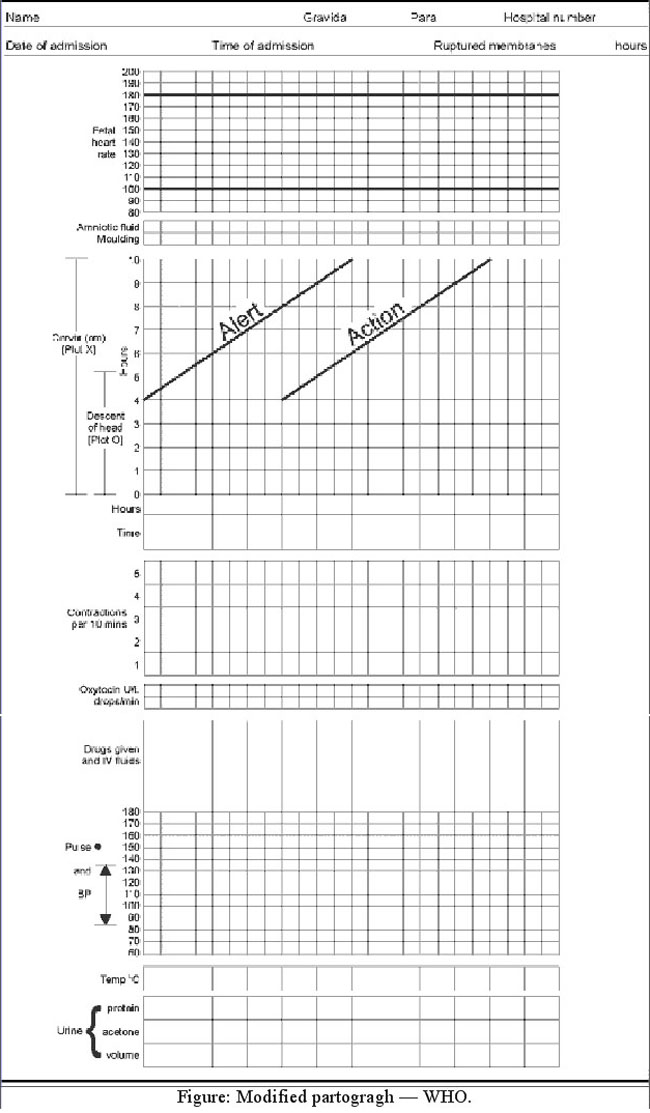

Supplement: S1 Fig — (TIF) [file pone.0172860.s001.tif]
