# Supplementary material for: Knowledge and utilization of the partograph: A cross-sectional survey among obstetric care providers in urban referral public health institutions in northwest and southwest Cameroon
Source: PLoS One. 2017 Feb 24;12(2):e0172860. doi: 10.1371/journal.pone.0172860 (PMC5325583; doi:10.1371/journal.pone.0172860)
Supplement: S1 File — (DOCX) [file pone.0172860.s002.docx]

Supplementary Material

Table I: Criteria for the partograph knowledge score (1).

|  |  | **Knowledge assessment** | **Scoring** |
| --- | --- | --- | --- |
| **1** |  | Correct definition of a partograph | **4** |
| **2** |  | Benefits derived from use of partograph |  |
|  | **i** | Reduce maternal mortality | **1** |
|  | **ii** | Reduce maternal morbidity | **1** |
|  | **Iii** | Reduce neonatal mortality | **1** |
|  | **iv** | Reduce neonatal morbidity | **1** |
|  | **v** | Increase efficiency in labour | **1** |
|  | **vi** | Mandatory for improved quality of care | **1** |
| **3** |  | Correct mention of the parts of a partograph |  |
|  | **i** | Fetal well-being | **1** |
|  | **ii** | Progress of labor | **1** |
|  | **iii** | Maternal well-being | **1** |
| **4** |  | Function of action line | **3** |
| **5** |  | Correct assessment of normal progress of labor | **3** |
| **6** |  | Components of labor assessment |  |
|  | **i** | Number of contractions | **1** |
|  | **ii** | Duration of contraction | **1** |
|  | **iii** | Duration of labour | **1** |
|  | **iv** | Time required to assess adequacy of contractions | **1** |
|  | **v** | Progress of labor | **1** |
|  |  | **Total** | **24** |

**Table II: Determinants of routine utilization of the partograph**

|  | **Not Routinely**  **N (%)** | **Routinely**  **N (%)** | **COR**  **(95% CI)** | ***p*-value** |
| --- | --- | --- | --- | --- |
| **Age** |  | | |  |
| ≤ 36 years | 27 (73.0) | 10 (27.0) | 1 |  |
| > 36 years | 21(61.8) | 13 (38.2) | 1.67 (0.61-4.55) | 0.315 |
| **Sex** |  | | |  |
| Male | 6 (60.0) | 4 (40.0) | 1 |  |
| Female | 42 (68.9) | 19 (31.1) | 0.68 (0.17-2.69) | 0.581 |
| **Marital Status** |  | | |  |
| Married | 29 (65.9) | 15 (34.1) | 1 |  |
| Single | 19 (70.4) | 8 (29.6) | 0.81 (0.29-2.29) | 0.697 |
| **Type of Institution** |  | | |  |
| R/D hospital | 21(61.8) | 13 (38.2) | 1 |  |
| MIWC | 27 (73.0) | 10 (27.0) | 0.59 (0.22-1.63) | 0.315 |
| **Professional Qualification** |  | | |  |
| Midwife | 19 (73.1) | 7 (26.9) | 1 |  |
| Nurse | 29 (64.4) | 16 (35.6) | 1.50 (0.52-4.32) | 0.455 |
| **Previous training** |  | | |  |
| Yes | 42 (65.6) | 22 (34.4) | 3.14 (0.82-8.08) | 0.303 |
| No | 6 (85.7) | 1 (14.3) | 1 |  |
| **Years of experience** |  | | |  |
| < 10 years | 24 (63.2) | 14 (36.8) | 1 |  |
| ≥ 10 years | 24 (72.7) | 9 (27.3) | 0.64 (0.23-1.77) | 0.392 |
| **Knowledge** |  | | | |
| Poor-to-fair | 32 (64.0) | 18 (36.0) | 1 | 0.320 |
| Good | 16 (76.2) | 5 (23.8) | 0.56 (0.17-1.77) |  |

**Reference**

1. Okokon I, Oku A, Agan T, Asibong U, Essien E, Monjok E. An Evaluation of the Knowledge and Utilization of the Partogragh in Primary, Secondary, and Tertiary Care Settings in Calabar, South-South Nigeria. International Journal of Family Medicine. 2014;http://dx.doi.org/10.1155/2014/105853.
