# Supplementary material for: Knowledge and utilization of the partograph: A cross-sectional survey among obstetric care providers in urban referral public health institutions in northwest and southwest Cameroon
Source: PLoS One. 2017 Feb 24;12(2):e0172860. doi: 10.1371/journal.pone.0172860 (PMC5325583; doi:10.1371/journal.pone.0172860)
Supplement: S3 File — (DOCX) [file pone.0172860.s004.docx]

**Knowledge and Utilisation of the Partograph among Obstetric Care Providers**

Code ……… Date …………………… Name of institution ……………………………………...

*Tick (✓) as applicable*

1. Age (years) ………………….
2. Sex: 1. Male ……. 2. Female …..
3. Religion: 1. Christian …….. 2. Muslim …….. 3. Others ……
4. Marital status: 1. Married …. 2. Single …. 3. Divorced …. 4. Widow …. 5. Widower ….
5. Professional qualification: 1.Midwife ….. 2. Nurse ……
6. Years of experience: ………
7. Have you seen a partograph before? 1. Yes ….. 2. No …….
8. Have you ever used a partograph before? 1. Yes ….. 2. No …….
9. Is the partograph available in your labour ward at the present time?

1. Yes ….. 2. No …. 3. I don’t know ……

1. Indicate how often you use the partograph for women in your labour ward:

1. Never …. 2. Rarely ….. 3. Sometimes ……. 4. Often …….. 5. Always …..

1. What is your reason (s) for not routinely using the partograph:

1. Time-consuming (much details to fill) …….. 2. Shortage of staff …… 3. Non-availability of partograph …… 4. Little or no knowledge on how to fill it ……… 4. It’s easier to manage labour without use of the partograph ……..

1. Did you receive any training on the use of partograph? 1. Yes ….. 2. No …….
2. The partograph may be defined as?

|  | Yes | No | Don’t know |
| --- | --- | --- | --- |
| A chart developed by midwives in developing countries to monitor labour |  |  |  |
| A complex tool with pictorial overview of labour for use by midwives |  |  |  |
| A chart for monitoring labour by doctors |  |  |  |
| A simple graphic recording of labour and salient conditions of the mother and foetus against time in hours |  |  |  |

1. Indicate your agreement with the following

| Using the partograph… | Yes | No | Don’t Know |
| --- | --- | --- | --- |
| Will reduce maternal mortality |  |  |  |
| Will reduce maternal morbidity |  |  |  |
| Will reduce neonatal mortality |  |  |  |
| Will reduce neonatal morbidity |  |  |  |
| Will increase the efficiency of those attending to women in labour |  |  |  |
| Is mandatory for improved quality of care of a woman in labour |  |  |  |

1. In a normal progress of labour;

|  | Yes | No | Don’t know |
| --- | --- | --- | --- |
| The graph/plot on the partograph should fall to the left of the alert line |  |  |  |
| The graph/plot on the partograph should fall on the alert line |  |  |  |
| The graph/plot on the partograph should fall to the right of the alert line |  |  |  |

1. The followings are functions of the action line on the Partograph

|  | Yes | No | Don’t know |
| --- | --- | --- | --- |
| Indicates appropriate action must be taken |  |  |  |
| Allows time for the woman to be adequately assessed for appropriate intervention |  |  |  |
| Continuous observation and monitoring till delivery |  |  |  |

1. During labour:

|  | Yes | No | Don’t Know |
| --- | --- | --- | --- |
| Three contractions in every 10 minutes is normal |  |  |  |
| Minimum duration of a strong contraction is 40 seconds |  |  |  |
| You require 10 minutes to effectively assess adequacy of contractions |  |  |  |
| Progress of labour is assessed by the degree of cervical dilatation and descent of the presenting part |  |  |  |
| Labour is prolonged when it lasts more than 12 hours |  |  |  |

1. Which of the following diagnosis can you make with the partograph?

|  | Yes | No | Don’t Know |
| --- | --- | --- | --- |
| Prolonged labour |  |  |  |
| Obstructed labour |  |  |  |
| Poor progress of labour |  |  |  |
| Inefficient uterine action |  |  |  |
| Suspected foetal distress |  |  |  |
| Abnormal foetal heart rate |  |  |  |
| Satisfactory progress of labour |  |  |  |
| Need for augmentation of labour with oxytocin |  |  |  |
| Need for caesarean section |  |  |  |
| Dehydration in the mother |  |  |  |
